# 2163 Polypharmacy and patterns of prescription medication use among cancer survivors

**DOI:** 10.1017/cts.2018.297

**Published:** 2018-11-21

**Authors:** Caitlin Murphy, Hannah Fullington, Carlos Alvarez, Simon C. Lee, Andrea Betts, David Haggstrom, Ethan Halm

**Affiliations:** University of Texas Southwestern Medical Center Dallas

## Abstract

OBJECTIVES/SPECIFIC AIMS: The population of cancer survivors is rapidly growing in the United States. Long term and late effects of cancer, combined with ongoing management of other chronic conditions, make cancer survivors particularly vulnerable to polypharmacy and its adverse effects. We examined patterns of prescription medication use and polypharmacy in a population-based sample of cancer survivors. METHODS/STUDY POPULATION: Using data from the Medical Expenditure Panel Survey (MEPS), we matched cancer survivors (n=5216) to noncancer controls (n=19,588) by age, sex, and survey year. We defined polypharmacy as using 5 or more unique medications. We also estimated proportion of respondents prescribed specific medications within therapeutic classes and total prescription expenditures. RESULTS/ANTICIPATED RESULTS: A higher proportion of cancer survivors were prescribed 5 or more unique medications (64.0%, 95% CI 62.3%–65.8%) compared with noncancer controls (51.5%, 95% CI 50.4%–52.6%), including drugs with abuse potential. Across all therapeutic classes, a higher proportion of newly (≤1 year since diagnosis) and previously (>1 years since diagnosis) diagnosed survivors were prescribed medications compared to controls, with large differences in central nervous system agents (65.8% vs. 57.4% vs. 46.2%), psychotherapeutic agents (25.4% vs. 26.8% vs. 18.3%), and gastrointestinal agents (31.9% vs. 29.6% vs. 22.0%). Specifically, nearly 10% of cancer survivors were prescribed benzodiazepines and/or opioids compared to about 5% of controls. Survivors had more than double prescription expenditures (median $1633 vs. $784 among noncancer controls). Findings persisted similarly across categories of age and comorbidity. DISCUSSION/SIGNIFICANCE OF IMPACT: Cancer survivors were frequently prescribed a higher number of unique medications and inappropriate medications or drugs with abuse potential, increasing risk of adverse drug events, financial toxicity, poor adherence, and drug-drug interactions. Adolescent and young adult survivors appear at increased risk of polypharmacy.

